# Single-Cell Transcriptomic Analysis Reveals Developmental Relationships and Specific Markers of Mouse Periodontium Cellular Subsets

**DOI:** 10.3389/fdmed.2021.679937

**Published:** 2021-08-12

**Authors:** Mizuki Nagata, Angel Ka Yan Chu, Noriaki Ono, Joshua D. Welch, Wanida Ono

**Affiliations:** 1 Department of Orthodontics, University of Texas Health Science Center at Houston School of Dentistry, Houston, TX, United States; 2 Department of Computational Medicine and Bioinformatics, Department of Computer Science and Engineering, University of Michigan, Ann Arbor, MI, United States; 3 Department of Diagnostic & Biomedical Sciences, University of Texas Health Science Center at Houston School of Dentistry, Houston, TX, United States

**Keywords:** mesenchymal progenitor cells, parathyroid hormone-related protein, dental follicle, periodontium, single cell analysis, mouse genetic models

## Abstract

The periodontium is essential for supporting the functionality of the tooth, composed of diversity of mineralized and non-mineralized tissues such as the cementum, the periodontal ligament (PDL) and the alveolar bone. The periodontium is developmentally derived from the dental follicle (DF), a fibrous tissue surrounding the developing tooth bud. We previously showed through *in vivo* lineage-tracing experiments that DF contains mesenchymal progenitor cells expressing parathyroid hormone-related protein (PTHrP), which give rise to cells forming the periodontal attachment apparatus in a manner regulated by autocrine signaling through the PTH/PTHrP receptor. However, the developmental relationships between PTHrP^+^ DF cells and diverse cell populations constituting the periodontium remain undefined. Here, we performed single-cell RNA-sequencing (scRNA-seq) analyses of cells in the periodontium by integrating the two datasets, i.e. PTHrP-mCherry^+^ DF cells at P6 and 2.3kb Col1a1 promoter-driven GFP^+^ periodontal cells at P25 that include descendants of PTHrP^+^ DF cells, cementoblasts, osteoblasts and periodontal ligament cells. This integrative scRNA-seq analysis revealed heterogeneity of cells of the periodontium and their cell type-specific markers, as well as their relationships with DF cells. Most importantly, our analysis identified a cementoblast-specific metagene that discriminate cementoblasts from alveolar bone osteoblasts, including *Pthlh* (encoding PTHrP) and *Tubb3*. RNA velocity analysis indicated that cementoblasts were directly derived from PTHrP^+^ DF cells in the early developmental stage and did not interconvert with other cell types. Further, CellPhoneDB cell-cell communication analysis indicated that PTHrP derived from cementoblasts acts on diversity of cells in the periodontium in an autocrine and paracrine manner. Collectively, our findings provide insights into the lineage hierarchy and intercellular interactions of cells in the periodontium at a single-cell level, aiding to understand cellular and molecular basis of periodontal tissue formation.

## INTRODUCTION

The periodontium is an important structure anchoring the tooth to the bone, which is composed of diversity of mineralized and non-mineralized tissues such as the cementum, the alveolar bone, the gingiva and the periodontal ligament (PDL). Development of the highly functional periodontal attachment apparatus requires a precise coordination of cell fates and differentiation of primitive mesenchymal cells. However, the mechanisms underlying how diverse types of cells in the periodontium – PDL cells, cementoblasts, and alveolar bone osteoblasts – are developmentally related one another and to their precursor cell populations remains largely undefined.

The dental follicle (DF), a sac-like fibrous tissue surrounding the developing tooth bud, contains mesenchymal progenitor cells that provide a cellular source for the periodontal attachment apparatus that is formed at the later stage (1). We previously demonstrated that DF cells expressing parathyroid hormone-related protein (PTHrP) function as mesenchymal progenitor cells that orchestrate proper formation of the periodontal tissue in a manner mediated by parathyroid hormone (PTH)/PTHrP receptor signaling (2-4). Also in our previous study, we characterized PTHrP^+^ DF cells using a single-cell RNA sequencing (scRNA-seq) approach and defined cellular heterogeneity of PTHrP^+^ DF cells. Single-cell RNA-seq analysis has been successfully applied to tooth-related cell types in the previous studies (2, 5-7). However, cellular heterogeneity of the cells in the periodontium and their developmental relationships with their earlier precursor cells are not yet dissected by scRNA-seq. Definitive answers at a single cell level would resolve long-standing debates regarding whether cementoblasts genuinely represent a cell type distinct from osteoblasts, which largely emanates from lack of *in vivo* cementoblast specific markers (8, 9).

In this study, we performed scRNA-seq analyses of cells in the periodontium, and computationally defined their relationships with their precursor cells. To this end, we integrated the two scRNA-seq datasets i.e. PTHrP-mCherry^+^ DF cells at P6 that we published previously [GSE120108], and Col1a1(2.3kb)-GFP^+^ cells at P25 including descendants of PTHrP^+^ DF cells, cementoblasts, osteoblasts and PDL cells that we newly generated for this study [GSE168450], using the LIGER algorithm (10). Our findings provide insights into the lineage hierarchy and intercellular interactions of cells in the periodontium at a single-cell level, aiding to understand cellular and molecular basis of periodontal tissue formation.

## MATERIALS AND METHODS

### Mice

*PTHrP-mCkerry/null* knock-in and *PTHrP-creER* bacterial artificial chromosome (BAC) mice have been described previously (2, 11). *Col1a1(2.3kb)-GFP* (JAX013134), *osteocalcin (Oc)-GFP* (JAX017469) and *Rosa26-CAG-loxP-stop-loxP-tdTomato* (Ai14: *R26R-tdTomato*, JAX007914) mice were acquired from the Jackson laboratory. *Scleraxis (Scx)-GFP* mice were kindly provided by Ronen Schweitzer (Oregon Health and Science University, OR, USA). All procedures were conducted in compliance with the Guidelines for the Care and Use of Laboratory Animals approved by the University of Michigan’s Institutional Animal Care and Use Committee (IACUC), protocol 8944 and 9496 (Ono). All mice were housed in a specific pathogen-free condition, and analyzed in a mixed background. Tamoxifen (0.25mg) was injected intraperitoneally into P3 mice. Mice were euthanized by over-dosage of CO_2_ or decapitation under inhalation anesthesia in a drop jar (Fluriso, Isoflurane USP, VetOne).

### Tamoxifen

Tamoxifen (T5648; Sigma-Aldrich) was mixed with 100% ethanol until completely dissolved. Subsequently, a proper volume of sunflower seed oil (Sigma S5007) was added to the tamoxifen-ethanol mixture and rigorously mixed. The tamoxifen-ethanol-oil mixture was incubated at 60°C in a chemical hood until the ethanol evaporated completely. The tamoxifen-oil mixture was stored at room temperature until use.

### Histology and Immunohistochemistry

Samples were dissected under a stereomicroscope (Nikon SMZ-800) to remove soft tissues, and fixed in 4% paraformaldehyde overnight at 4°C, then decalcified in 15% EDTA for 7 days. Decalcified samples were cryoprotected in 30% sucrose/PBS solutions and then in 30% sucrose/PBS:OCT (1:1) solutions, each at least overnight at 4°C. Samples were embedded in an OCT compound (Tissue-Tek, Sakura) under a stereomicroscope and transferred on a sheet of dry ice to solidify the compound. Embedded samples were cryosectioned at 16μm using a cryostat (Leica CM1850) and adhered to positively charged glass slides (Fisherbrand ColorFrost Plus). Cryosections were stored at −20°C in freezers until use. Sections were postfixed in 4% paraformaldehyde for 20 min at room temperature. For immunostaining, sections were permeabilized with 0.25% TritonX/TBS for 30 min, blocked with 3% BSA/TBST for 30 min, and incubated with rabbit anti-TUBB3 polyclonal antibody (1:500, Abcam ab18207), or sheep anti-DMP1 polyclonal antibody (1:100, R&D AF4386) overnight at 4°C, and subsequently with Alexa Fluor 647-conjugated donkey anti-rabbit IgG (A31573) for 3 hours at room temperature. Sections were further incubated with DAPI (4’,6-diamidino-2-phenylindole, 5 μg/ml, Invitrogen D1306) to stain nuclei. *In situ* hybridization was performed with RNAscope 2.5 HD Reagent kit Brown (Advanced Cell Diagnostics 322300) using Foxf1 probe (Advanced Cell Diagnostics 473051) according to the manufacturer’s protocol. Stained samples were mounted in TBS with No.1.5 coverslips (Fisher).

### Imaging

Images for fixed sections and live cell culture were captured by an automated inverted fluorescence microscope with a structured illumination system (Zeiss Axio Observer Z1 with ApoTome.2 system) and Zen 2 (blue edition) software. The filter settings used were: FL Filter Set 34 (Ex. 390/22, Em. 460/50 nm), Set 38 HE (Ex. 470/40, Em. 525/50 nm), Set 43 HE (Ex. 550/25, Em. 605/70 nm), Set 50 (Ex. 640/30, Em. 690/50 nm) and Set 63 HE (Ex. 572/25, Em. 629/62 nm). The objectives used were: Fluar 2.5×/0.12, EC Plan-Neofluar 5×/0.16, Plan- Apochromat 10x/0.45, EC Plan-Neofluar 20×/0.50, EC Plan-Neofluar 40×/0.75, Plan- Apochromat 63×/1.40. Images were typically tile-scanned with a motorized stage, Z-stacked and reconstructed by a maximum intensity projection (MIP) function. Differential interference contrast (DIC) was used for objectives higher than 10×. Representative images of at least three independent biological samples are shown in the figures.

### Cell Preparation

Gingival tissues of detached mandibles were completely removed using sharp forceps, and dentoalveolar components including molars, dental sacs or periodontal tissue, were carefully resected using a disposable scalpel (No.15, Graham-Field). Molars (M1 and M2) were carefully extracted from sockets in a 35 mm dish containing 3 ml Ca^2+^, Mg^2+^-free Hank’s Balanced Salt Solution (HBSS, Sigma H6648) containing 2 Wunsch units of Liberase TM (Roche), and incubated at 37±C for 15 min. on a shaking incubator (ThermomixerR, Eppendorf). DF cells or periodontal cells were obtained by rigorous pipetting and filtration through a 70 μm cell strainer (BD) into a 50 ml tube on ice to make single cell suspension. Cells were pelleted and resuspended in appropriate medium for subsequent purposes.

### Single Cell RNA Sequencing (scRNA-seq) Analysis of FACS-Sorted Periodontal Cells

Dissociated periodontal cells harvested from P25 *Col1(2.3kb)*-GFP;*PTHrP-creER;R26R*-tdTomato molars were pooled. Cell sorting was performed using a six-laser Sony Synergy SY3200 (Ex.350/405/488/561/594/685nm) high-speed cell sorter with a 100-μm nozzle. *Col1a1(2.3kb)*-GFP^+^ cells including tdTomato^+^ cells were directly sorted into ice-cold DPBS/1% FBS, pelleted by centrifugation and resuspended in 30 μl DPBS/1% FBS using wide-bore pipettes. Cell numbers were quantified by Countless II automated Cell Counter (ThermoFisher) before loading onto the Chromium Single Cell 3’ microfluidics chip (10× Genomics Inc., Pleasanton, CA). cDNA libraries were sequenced by Illumina HiSeq 4,000 using two lanes and 50 cycle paired-end read, generating a total of ~ 770 million reads. The sequencing data was first pre-processed using the 10× Genomics Cell Ranger software. For alignment purposes, we generated and used a custom genome fasta and index file by including the sequences of mCherry to the mouse genome (mm10). The scRNA-seq dataset presented herein have been deposited in the National Center for Biotechnology Information (NCBI)’s Gene Expression Omnibus (GEO) and are accessible through GEO Series accession number GSE120108 and GSE168450. The dataset GSE120108 has been published previously (2).

### Integrative Single-Cell Computational Transcriptomic Analysis

We filtered out cells with <1,000 genes per cell and with more than 15% mitochondrial read content.

#### LIGER Data Integration

LIGER (version 0.5.0) (10) was used for sample integration, normalization, clustering, and visualization. A joint LIGER object was created using the two datasets (*PTHrP-mCherry* and *Col1a1(2.3kb)-GFP*). We followed our recently published protocol (12). Gene expression count data for the combined sample was normalized and scaled with functions “normalize” and “scaleNotCenter”. Next, we selected 3,000 highly variable genes across samples with function “selectGenes”. We utilized function “optimizeALS” for integrative non-negative matrix factorization (iNMF) and function “quantile_norm” for quantile alignment. Two-dimensional visualization and clustering were carried out with “louvainCluster” at resolution 0.75 and “runUMAP” with cosine distance, nearest neighbors set to 30, and minimum distance to 0.55. While *Ptprc*^+^ hematopoietic cells, *Cdh5*^+^ endothelial cells, *Krt5*^+^ epithelial cells, and *Plp1*^+^ glial cells were filtered, mesenchymal cells, expressing a high level of *Postn*, were retained (Supplementary Figure 1B). The *Postn*^+^ cells underwent re-normalization and re-clustering with similar procedures as described, with the exception that “louvainCluster” was applied using a resolution of 0.90 and “runUMAP” using a minimum distance of 0.45.

To identify the differentially expressed genes (DEGs) among clusters, non-parametric Wilcoxon rank sum test was performed, with function “runWilcoxon”. We filtered statistically significant (adjusted *p*-value < 0.05) and highly differentially expressed (logFC > 3) genes for clusters, with the exception of cluster 6, the DEGs of which were discovered by setting the logFC threshold to 1.5. The top 100 DEGs were selected for comparison and were used for cluster annotation. Being distinguished as macrophages (*Cd86*+ and *Itgam*+), cells in cluster 19 were filtered. Characterized by known molecular markers, cells were assigned to DF cells, fibroblasts, PDL cells, transitional cells, cementoblasts, DP cells, osteoblasts, and marrow stromal cells (Supplementary Figure 2). Cluster identities were demonstrated in Figure 2B. A total of 2,738 filtered cells were used for further scRNA downstream analysis.

#### RNA Velocity Analysis (velocyto and scVelo)

The two sample-specific aligned bam files were used as input for velocyto (13) to quantify the unspliced and spliced abundances in loom format, and subsequently were merged with function “adata.concatenate”. To calculate the RNA velocities (rates of transcription, splicing and degradation) of the single cells, we utilized scVelo (version 0.2.2) (14), exploring future cell trajectories. The combined loom file was normalized and log-transformed with function scvelo.pp.filter_and_normalize(min_shared_counts = 20, n_top_genes = 5,500, flavor = “seurat”). The first and second order moments for each cell across its nearest neighbors were computed with function scvelo.pp.moments(method = ‘umap’, n_neighbors = 30, n_pcs = 30, knn = True). The velocities were estimated by running the likelihood-based dynamical model with function scvelo.tl.velocity(mode = “dynamical”) and the velocity graph was constructed with scvelo.tl.velocity_graph(). To test for the presence of differential kinetics across cell types that could not be well explained by a single model of the overall dynamics, we applied the function scvelo.tl.differential_kinetic_test. RNA velocities were then recomputed with functions scvelo.pp.neighbors(method = ‘umap’, n_neighbors = 30, n_pcs = 30, knn = True), scvelo.tl.velocity(diff_kinetics = True), and scvelo.tl.velocity_graph(). To visualize the RNAvelocities, the cluster assignments and UMAP coordinates were extracted from the LIGER analysis output. Velocities were projected onto the UMAP coordinates, using function scvelo.tl.velocity_embedding_stream(basis = ‘umap’). The resulting visualization can be found in Figure 3A.

### CellPhoneDB, Intercellular Communication Analysis

To predict enriched ligand-receptor relationships between cementoblasts and other cell populations, we performed CellPhoneDB analysis (15). CellPhoneDB v.2.0 was employed with default parameters. Interactions of *Pthlh-Pth1r* and *Wnt7b-Fzd4* were displayed in Figure 3C.

## RESULTS

### Fluorescent Transgenic Marking of PTHrP^+^ DF Cells and Col1a1^+^ Periodontal Cells

**FIGURE 1 ∣:**
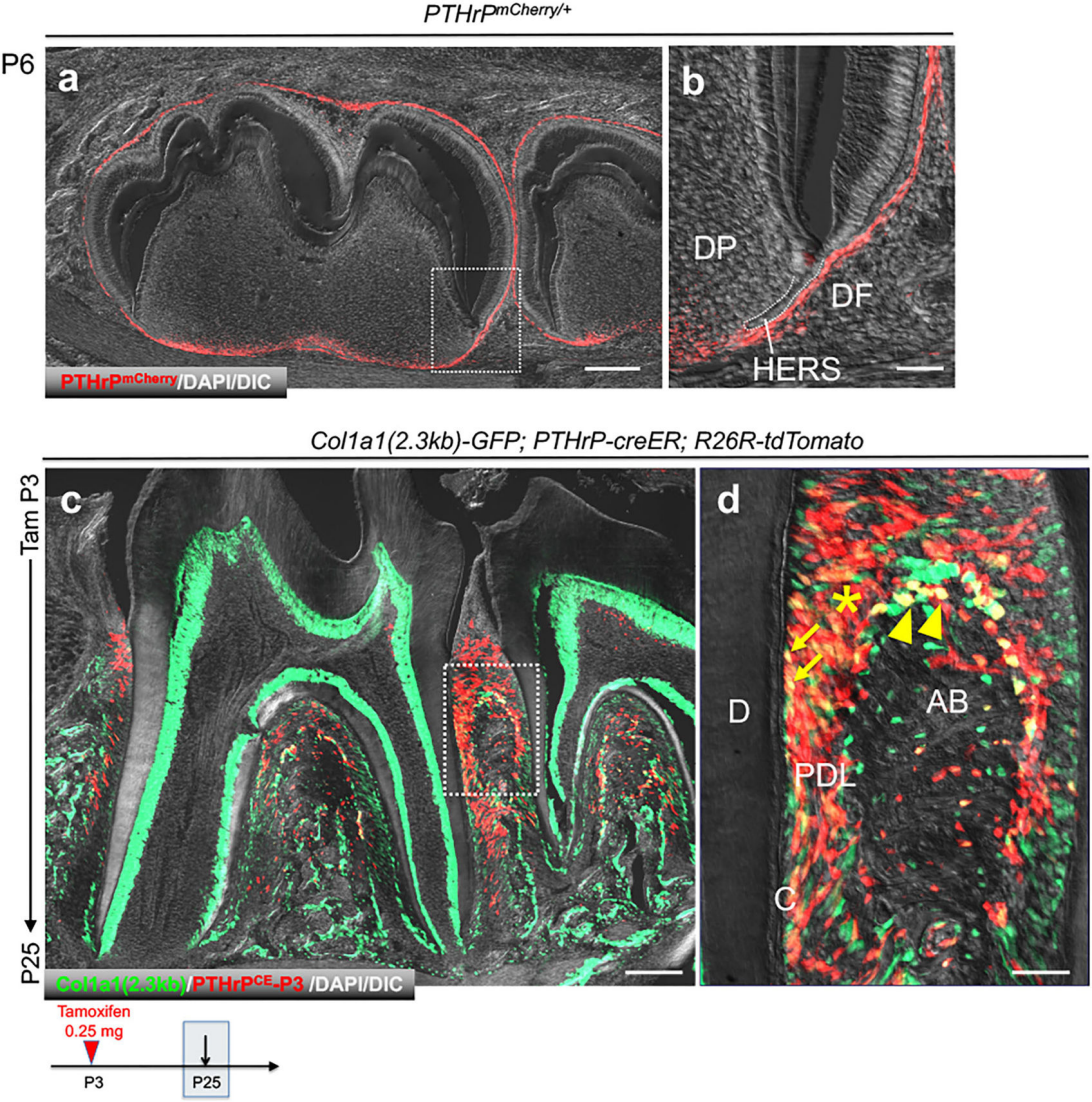
PTHrP^+^ DF mesenchymal progenitor cells differentiate into Col1a1-GFP^+^ periodontal cells during tooth root development. **(a,b)** Mandibular tooth bud of *PTHrP*^mCherry/+^. DP: dental papilla, DF; dental follicle, HERS: Hertwig’s epithelial root sheath. **(c,d)** Mandibular molars of *Col1a1(2.3kb)-GFP*; *PTHrP-creER;R26R*-tdtomato. D: dentin, C: cementoblast, PDL: periodontal ligament, AB: alveolar bone. Arrow: Col1a1-GFP^+^tdTomato^+^ cementoblasts on the root surface, Arrowhead: Col1a1-GFP^+^tdTomato^+^ osteoblasts on the cryptal alveolar bone, *: Col1a1-GFP^+^tdTomato^+^ PDL cells. Scale bars: 200 μm **(a,c)**, 50 μm **(b,d)**.

We first set out to define a fluorescent transgenic mouse model that marks cells in the periodontium. We previously reported an scRNA-seq dataset of PTHrP-mCherry^+^ DF cells at P6 [GSE120108] that were harvested from *PTHrP-mCherry* knock-in mice using fluorescence activated cell sorting (FACS) (2). Histologically, PTHrP-mCherry^+^ cells were predominantly found in the DF surrounding the developing tooth at P6 (Figures 1a,b). Subsequently, we analyzed *Col1a1(2.3kb)-GFP*; *PTHrP-creER*; *R26R-tdTomato* mice at P25 [GSE168450], which were treated with tamoxifen at P3, when the tooth root begins to develop. In these compound transgenic mice, cells with 2.3kb Col1a1 promoter activities express GFP, whereas descendants of PTHrP^+^ DF cells at P3 express tdTomato. At P25, when formation of the tooth root and the periodontium was complete, Col1a1-GFP was broadly expressed by cells in the periodontium, including those in the dentin, the cementum on the root surface, PDL, and the alveolar cryptal bone (Figures 1c,d). As we reported previously, tdTomato^+^ cells were localized at the acellular cementum, PDL and the alveolar cryptal bone; and a majority of tdTomato^+^ cells were also positive for Col1a1-GFP. Therefore, Col1a1-GFP marks diverse array of cells in the periodontium, including descendants of PTHrP^+^ DF cells.

### scRNA-seq Analysis Reveals Fundamental Cellular Heterogeneity of the Mouse Periodontium

**FIGURE 2 ∣:**
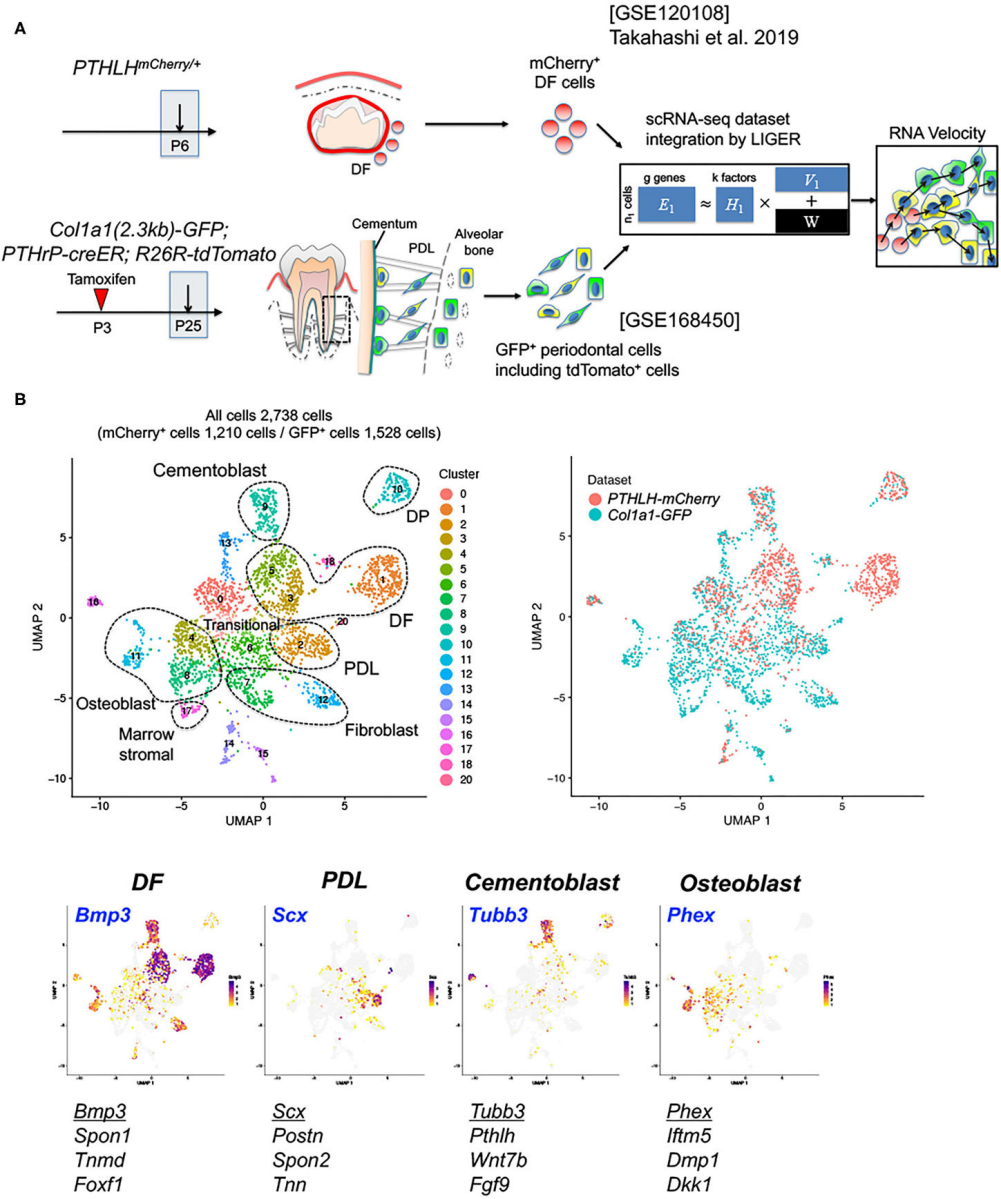
Characterization of PTHrP-mCherry^+^ DF cells and Col1a1-GFP^+^ periodontal cells. **(A)** Overview of the scRNA-seq analysis workflow. PTHrP-mCherry^+^ cells at P6 and Col1a1-GFP^+^ periodontal cells at P25 were harvested, and LIGER takes two single-cell datasets, GSE120108 and GSE168450 as input. We integrated single-cell datasets using LIGER and performed RNA velocity analysis. **(B)** UMAP-based visualization of major classes of FACS-sorted cells; dots indicate individual cells. Left upper panel: cells colored by LIGER clustering. Right upper panel: cells colored by sample. Feature plot of cell-type specific markers. High expression: violet, Low expression: yellow, No expression: gray. A total of 2,738 filtered cells from two datasets, 1,210 PTHrP-mCherry^+^ cells and 1,528 Col1a1-GFP^+^ cells. DF: dental follicle, DP: dental pulp, PDL: periodontal ligament.

Next, we set out to define cellular heterogeneity of Col1a1-GFP^+^ cells in the periodontium and their developmental relationships with PTHrP^+^ DF cells using integrative scRNA-seq analyses. For this purpose, we isolated Col1a1-GFP^+^ cells by FACS-sorting from the periodontium of mandibular molars of *Col1a1(2.3kb)-GFP*; *PTHrP-creER*; *R26R-tdTomato* at P25 after a tamoxifen pulse at P3 (Supplementary Figure 1A). Total of 4,578 cells were profiled by the 10X Chromium Single-Cell Gene Expression Solution platform. Subsequently, this P25 Col1a1-GFP^+^ periodontal dataset was integrated with the previously published PTHrP^+^ DF dataset using LIGER (Figure 2A) (10). We discovered 24 clusters (Cluster 0–23) among total of 8,630 cells, which included mesenchymal cells as well as contaminating hematopoietic cells, endothelial cells, epithelial cells and other cells (Supplementary Figure 1B). We subsequently focused on mesenchymal cell populations that highly expressed *Postn* (Cluster 1–3, 5, 8, 14, 17, 19), by eliminating contaminating cells, and re-clustered these cells into 21 clusters (Figure 2B). These clusters included cells expressing *mCherry*, *eGFP* and *tdTomato*, wherein their expression patterns were consistent with the distribution of these two datasets at two different time points of P6 and P25 (Supplementary Figure 2). We manually defined 7 major cell populations, including DF cells expressing *Bmp3* (16) and *Spon1* (17) (Cluster 1, 3 and 5), PDL cells expressing *Scx* (18) and *Postn* (19) (Cluster 2), cementoblasts expressing *Tubb3* (20) and *Pthlh* (encoding PTHrP) (2) (Cluster 9), DP cells expressing *Tac1* (5) (Cluster10), osteoblasts expressing *Phex* (21) and *Ifitm5* (22) (Cluster 4, 8 and 11), fibroblasts expressing *S100a4* (23) (Cluster 7 and 12), marrow stromal cells expressing *Ebf3* (24) (Cluster 17) (Figure 2B, Supplementary Figure 2). Interestingly, cells in cluster 0 and 6 exhibited transitional states between PDL cells and osteoblasts. Subsequently, we validated some of the identified marker genes *in vivo*. As expected, *Foxf1* was expressed by DF cells, Scx-GFP was expressed by PDL cells, and DMP1 was expressed in the alveolar cryptal bone osteoblasts (Supplementary Figures 3a-c). Therefore, these scRNA-seq analyses reveal cellular heterogeneity of Col1a1^+^ cells in the periodontium, and their relationships with PTHrP^+^ DF cells.

### Computational Analysis of RNA Velocity and Intercellular Communications Among Periodontal Cellular Subsets

**FIGURE 3 ∣:**
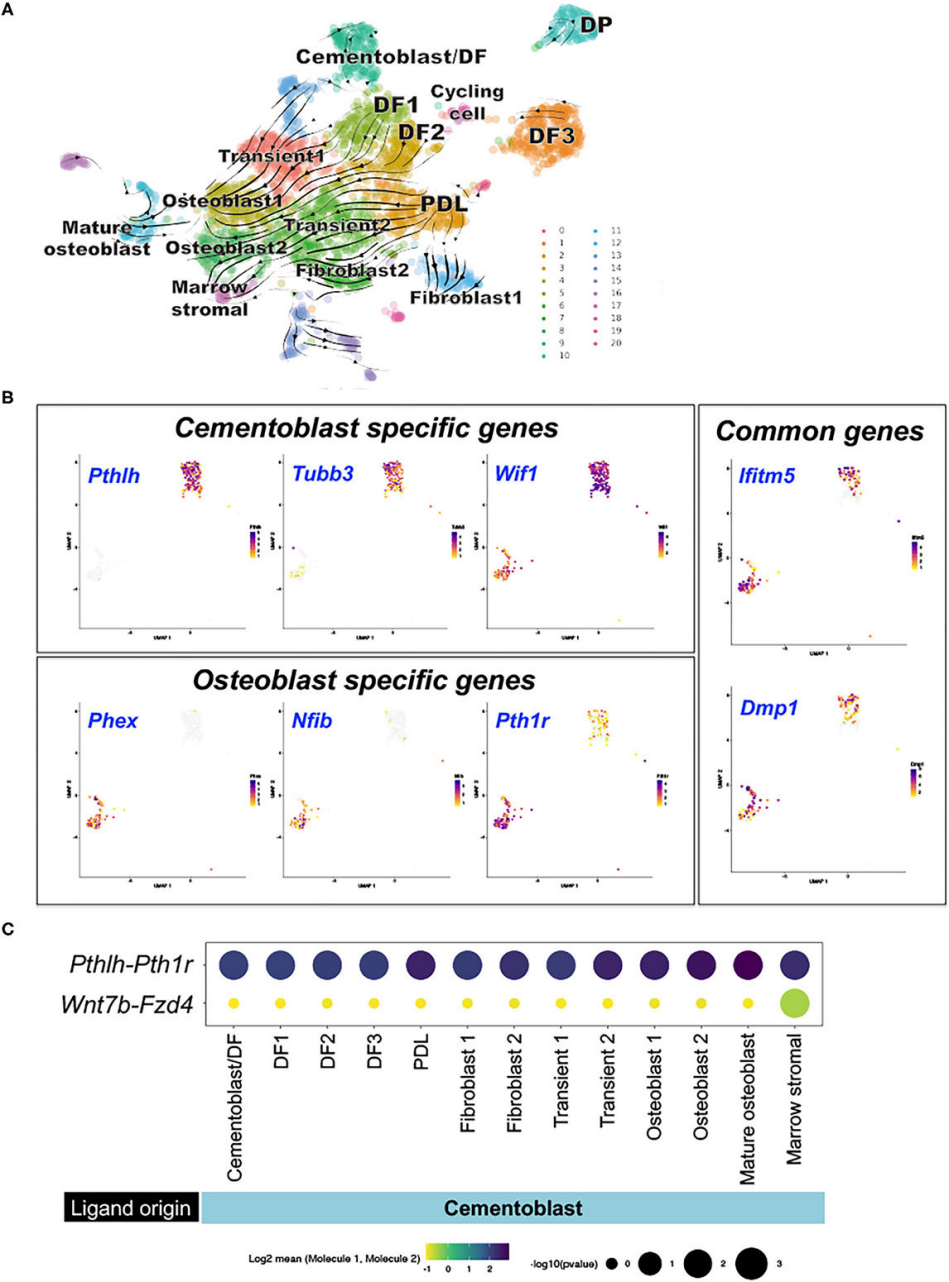
Computational lineage inference and intercellular communications of subset of PTHrP-mCherry^+^ DF cells and Col1a1-GFP^+^ periodontal cells. **(A)** RNA velocity analysis. Left panel: Dynamic velocity vectors superimposed on UMAP plot. Arrows: dynamic velocity vectors predicted future cell states. Right panel: cells colored by sample, **(B)** Comparison DEGs between cluster 9 and 11. Wilcoxon signed-rank test was conducted to identify differentially expressed genes. High expression: violet, Low expression: yellow, No expression: gray. **(C)** CellPhoneDB intercellular communication analysis. Split-dot-based visualization of ligand-receptor interactions among cementoblast and other cell subsets. Top row: *Pthlh-Pth1r*, bottom row: *Wnt7b-Fzd4* pairs. Columns: ligand-expressing (bottom) and receptor- expressing (top) clusters. DEGs: differentially expressed genes, DF: dental follicle, DP: dental pulp, PDL: periodontal ligament.

To identify the developmental relationships among cells in the periodontium, PTHrP^+^ DF cells and their descendants, we subsequently analyzed the integrated dataset with RNA velocity, which is a computational approach that infers whether genes are actively being up- or down-regulated in individual cells based on the ratio of spliced to unspliced transcripts (13, 14). This analysis predicts the routes by which cell populations develop into each other. RNA velocity analysis predicted three putative points of origins, including the two major subgroups of DF cells, DF1 and DF2, and Scx^+^ PDL cells (Figure 3A). DF1 and DF2 cells developed into osteoblasts through transitional cell populations, reflecting the expected differentiation cascade during formation of the periodontium (Figure 3A). In addition, Scx^+^ PDL cells also developed into osteoblasts and marrow stromal cells through transitional cell populations, indicating that Scx^+^ PDL cells may contain a population of mesenchymal stem cells.

We also found another major DF cell population, DF3, which was not closely related to other periodontal cell populations, indicating this subset of PTHrP^+^ DF cells might not contribute to development of periodontium. Interestingly, a group of PTHrP^+^ DF cells overlapped extensively with a group of P25 Col1a1^+^ cells (Cementoblast/DF); these cells simultaneously expressed mature osteoblast markers *Ifitm5* and *Dmp1* and cementoblast markers *Tubb3* and *Pthlh*. These cementoblasts did not interconvert with other cell populations, indicating that cementoblasts diverge from other PTHrP^+^ DF cells early during formation of the periodontium.

We further capitalized on this integrated dataset and defined a cementoblast-specific “metagene”, some of which may serve as cementoblast-specific marker genes. The cementum and the bone are similar mineralized tissues; however, the cementum has its unique anatomical feature and functionality (25). Currently, no specific marker gene is available to distinguish cementoblasts from osteoblasts (8, 9). To identify cementoblast-specific markers, we analyzed the top 20 genes that are differentially expressed in cementoblasts (Cluster 9) or mature osteoblasts (Cluster 11) (Supplementary Figure 4). We confirmed that several previously reported cementoblast markers, such as *Wif1* (9) and *Tubb3* (20), were enriched in cluster 9; we also confirmed that *Pthlh* (encoding PTHrP) was highly expressed in these cells. In contrast, *Phex*, *Nfib* and *Pth1r* were highly expressed in cluster 11 corresponding to mature osteoblasts (Figure 3B). We also confirmed that the representative osteoblast marker genes, *Ifitm5* and *Dmp1*, were expressed in cells of both cluster 9 and 11, denoting their fundamental characteristics as mineralizing cell types. Therefore, these analyses successfully identified a panel of genes that could serve as novel specific marker metagene for cementoblasts.

We further analyzed the dataset with CellPhoneDB (15), a computational analysis that uses cell-type-specific expression of annotated ligand-receptor gene pairs to quantify the evidence for signaling interactions between cell types. We focused on the ligand-receptor pairs that involve cementoblasts, particularly *Pthlh* (encoding PTHrP) and *Wnt7b* that are abundantly expressed by cementoblasts. *Pthlh-Pth1r* interactions were evident across a broad range of cells, including among cementoblasts and between cementoblasts and PDL cells, osteoblasts and marrow stromal cells (Figure 3C). In contrast, *Wnt7b-Fzd4* interactions were only notable between cementoblasts and marrow stromal cells (Figure 3C). Therefore, these findings indicate that PTHrP secreted by cementoblasts interact with PTH/PTHrP receptor expressed by cementoblasts and their surrounding cells, such as PDL cells and osteoblasts, both in an autocrine and paracrine manner.

### PTHrP Specifically Marks Cementoblasts, but Not Alveolar Bone Osteoblasts

**FIGURE 4 ∣:**
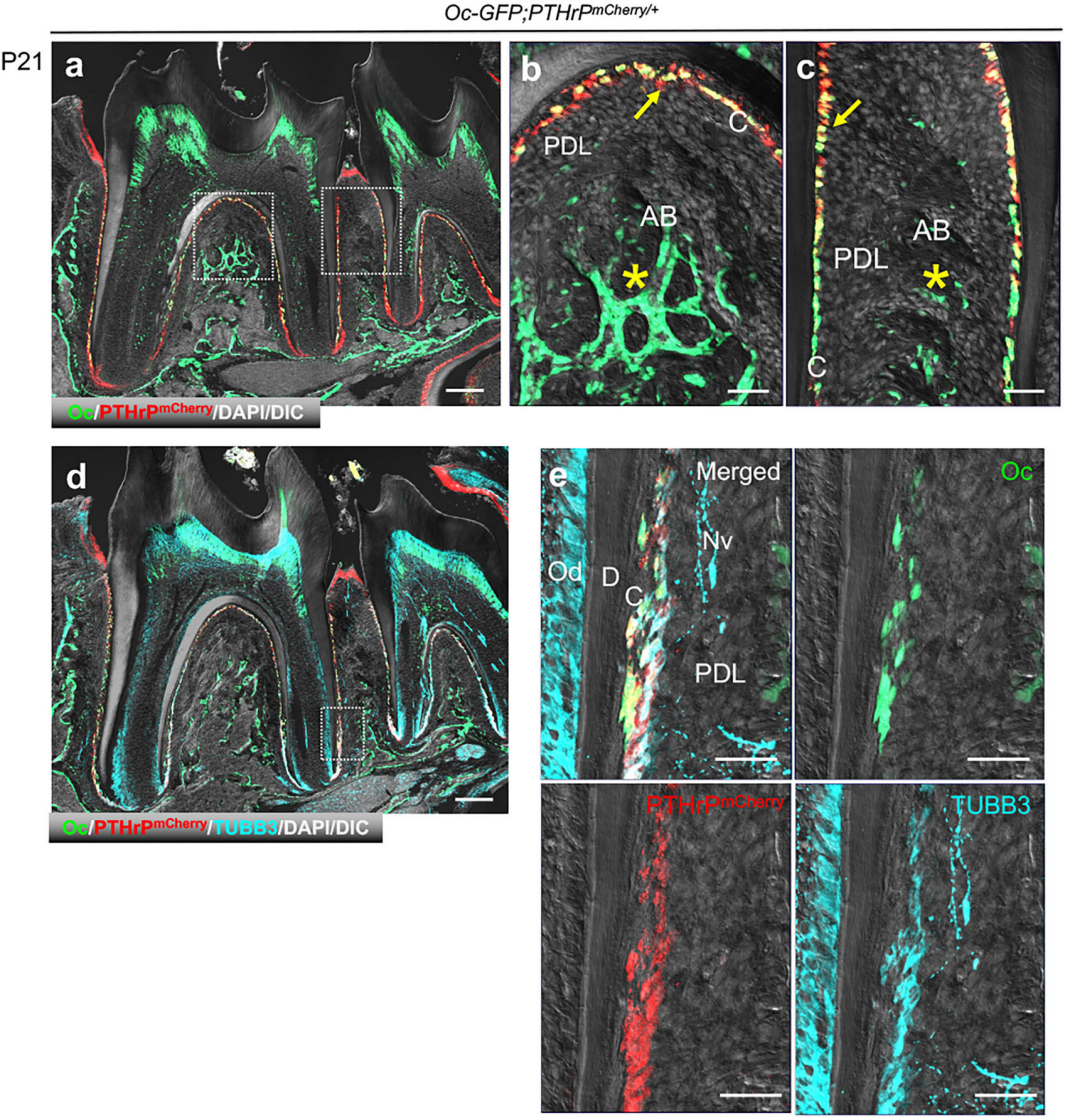
PTHrP is expressed by cementoblasts, but not osteoblasts. **(a–e)** Mandibular molars of *Osteocalcin(Oc)-GFP;PTHrP*^mCherry^. C: cementoblast, PDL: periodontal ligament, AB: alveolar bone, Nv: nerve, D: dentin, Od: odontoblast. Arrow: PTHrP-mCherry^+^Oc-GFP ^+^ cementoblasts, *: Oc-GFP^+^ osteoblasts. Scale bars: 200 μm **(a,d)**, 50 μm **(b,c,e)**.

Lastly, we set out to test the validity of the cementoblast-specific markers that we identified in our scRNA-seq analyses. First, we examined the *in vivo* expression pattern of PTHrP using *PTHrP-mCherry*; *Osteocalcin (Oc)-GFP* mice. Osteocalcin (Oc)-GFP specifically labels cementoblasts and osteoblasts, but not PDL fibroblasts (9). At P21, PTHrP-mCherry was co-expressed by Oc-GFP^+^ cementoblasts, but not by Oc-GFP^+^ osteoblasts in the alveolar cryptal bone (Figures 4a-c). We further examined β3-tublin (TUBB3) expression in PTHrP-mCherry^+^ cementoblasts using immunohistochemistry. TUBB3 was expressed in a similar pattern with PTHrP-mCherry, while Oc-GFP was more restricted to the cementoblast layer (Figures 4d,e). In addition, while PTHrP-mCherry was specifically expressed by cementoblasts, TUBB3 was also expressed by odontoblasts and the neural bundle in the PDL space. These findings indicate that PTHrP can serve as a novel specific marker of cementoblasts.

## DISCUSSION

Taken together, our findings provide insights into the lineage hierarchy and intercellular interactions of cells in the periodontium at a single-cell level, aiding to understand cellular and molecular basis of periodontal tissue formation. Single-cell RNA-sequencing analysis is a powerful approach to unravel cellular heterogeneity of a target cell population, and has been applied to dental cell types (2, 5). In this study, we revealed in-depth heterogeneity of cells in the periodontium and their developmental relationships with PTHrP^+^ DF cells at a single-cell level, by integrative scRNA-seq analysis of fluorescently isolated cells of interest. After eliminating contaminating cells such as hematopoietic, endothelial and epithelial cells from the dataset, we found that PTHrP^+^ DF cells can be classified into three subtypes in terms of their relationships with osteoblasts and cementoblasts. Biological studies demonstrate that DF plays an important role for tooth eruption and tooth root formation (2, 26). These results suggest that PTHrP^+^ DF cells are composed of distinct populations of precursor cells with pre-determined cell fates to osteoblasts and cementoblasts, which contribute differentially to tooth root formation and tooth eruption. It is interesting that PTHrP^+^ DF cells did not show a direct lineage contribution to *Scx*-expressing PDL cells in our dataset. PDL is a fibrous connective tissue that connects the tooth to the alveolar bone and possesses the stem/progenitor cell populations for periodontal wound healing and homeostasis (27-29). Scleraxis (Scx) is a basic helix-loop-helix transcriptional factor that is abundantly expressed by tendons and ligaments (30), and also predominantly expressed in mature PDL fibroblasts and acts as a negative regulator of PDL mineralization (18). Based on RNA velocity analysis, Scx^+^ PDL cell population (Cluster 2) appears to provide a cellular origin for osteoblasts and other fibroblasts, indicating that Scx may serve as a marker of periodontal ligament stem cells (PDLSCs). Men et al. recently reported Gli1^+^ cells in periodontium function as PDLSCs *in vivo* (31). Our computational analysis showed that *Gli1* is expressed by cells in DF, DP and transient state, but not in cluster 2 (Supplementary Figure 2), suggesting Gli1^+^ cells may mark transitional mesenchymal precursors in PDL. However, additional *in vivo* study will be needed to reveal the bona fide marker of PDLSC *in vivo*.

The cementum is a thin mineralized tissue that covers the entire root surface, which is one of the three structures that support the tooth in concert with the PDL and the alveolar bone (25). Functional regeneration of the periodontium largely depends on the regenerative potential of the cementum on the root surface; however, mechanisms of cementum development are largely unknown (8). Importantly, our RNA velocity analysis clearly predicted different routes for cementoblasts and osteoblasts. More specifically, we observed a continuous predicted lineage hierarchy from PTHrP^+^ DF cell subsets or Scx^+^ PDL cells to osteoblasts, whereas cementoblasts appeared to constitute their own “mini-lineage” without interconverting with other cell types, indicating precursor cells for cementoblasts have distinct characteristics from those of osteoblasts. Recently, Zhao et al. also reported the single-cell computational analysis of mesenchymal cell populations in the periodontium, and showed that CD90^+^ cells could differentiate into cementoblasts (32). In contrast, our computational analysis did not detect upregulation of *Axin2* or *Thy1* (encoding CD90) in cementoblasts. Moreover, Zhao et al. predicted that cementoblast/osteoblast clusters appear to contain less terminally differentiated cells as compared to the mesenchymal cells expressing *Postn*, whereas our analysis demonstrated that osteoblasts present as terminally differentiated cell populations. One possible reason for this discrepancy may be related to the difference in the experimental design. Zhao et al. isolated bulk periodontal cells from CD-1 mice after tooth root formation prior to profiling. In contrast, we isolated Col1a1-GFP^+^ periodontal cells by FACS-sorting prior to single-cell transcriptomic analysis, and further integrated the dataset with that of PTHrP-mCherry^+^ DF cells. Further studies are needed to define the lineage hierarchy of cells in the periodontium in a more definitive manner.

**FIGURE 5 ∣:**
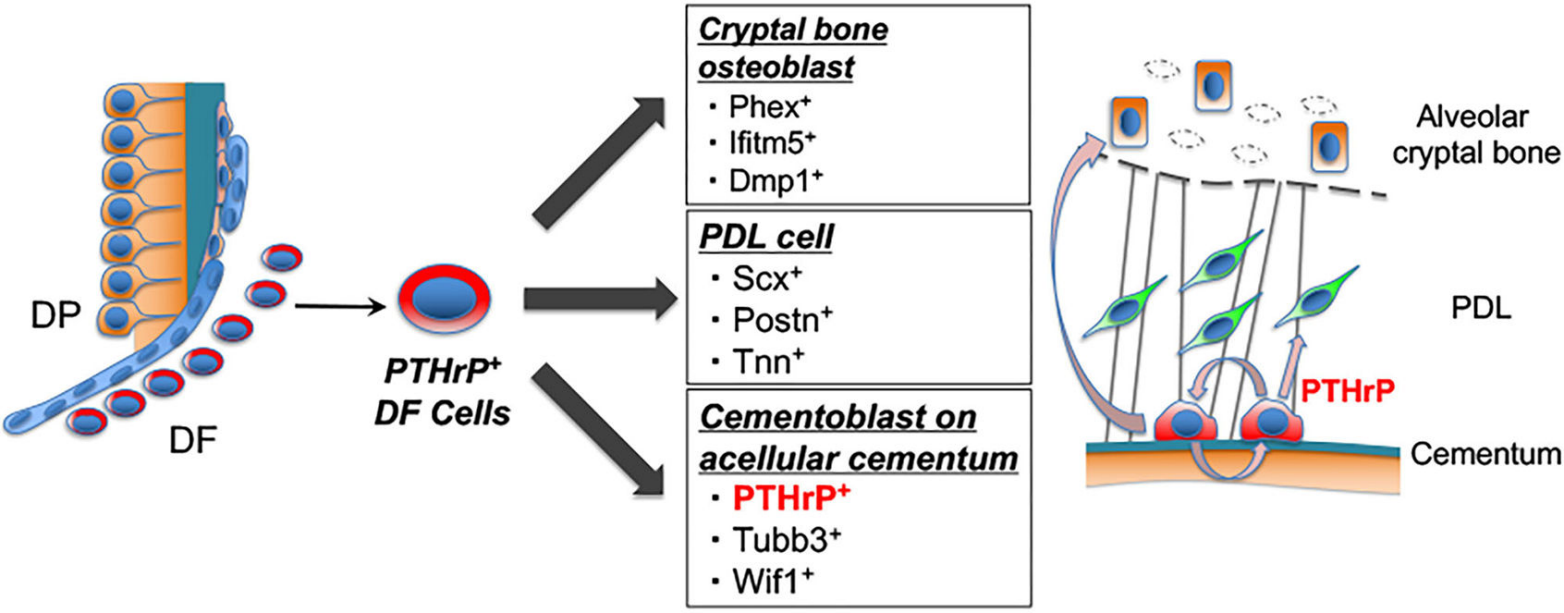
PTHrP^+^ DF cells contribute to attachment apparatus formation and PTHrP is a specific marker for cementoblast. Shown is the concluding diagram of this study. PTHrP^+^ DF cells give rise to osteoblasts, PDL cells, cementoblasts. After attachment apparatus formation, PTHrP is specifically expressed in cementoblasts. Our computational analysis suggests that PTHrP secreted from cementoblasts is important for cementogenesis via PTH/PTHrP receptor signaling.

PTHrP is a locally acting autocrine/paracrine ligand, and plays an important role in the formation of skeletal tissues via signaling by its receptor, PTH/PTHrP receptor (33). In postnatal growth plates, the resting zone harbors PTHrP^+^ skeletal stem cells that maintain the integrity of the growth plate (11). In tooth development, PTHrP is abundantly expressed in DF surrounding the developing tooth. We found that PTHrP is expressed in cementoblasts on the entire root surface after tooth root formation. It has been reported that PTHrP regulates cementogenesis by repressing mRNA expression of *bone sialoprotein* (*Bsp*) and *Oc in vitro* (34). Indeed, our CellPhoneDB analysis predicted that PTHrP secreted from cementoblasts interacts with PTH/PTHrP receptor expressed by diversity of other cell populations, whereas Wnt7b secreted from cementoblasts interacts with Fzd4, a receptor of Wnt ligand, specifically on marrow stromal cells. Moreover, PTHrP is specifically expressed in cementoblasts, whereas TUBB3 is expressed not only in cementoblasts but also in the terminal nerve in the PDL space and odontoblasts of the radicular dentin (Figures 4d,e). These findings indicate that PTHrP can serve as a bona fide marker for cementoblasts that allows us to distinguish cementoblasts from alveolar bone osteoblasts, while serving as an important regulator of cementogenesis (Figure 5).

In conclusion, our integrative scRNA-seq analyses provide insights into the lineage hierarchy and intercellular interactions of cells in the periodontium at a single-cell level, aiding to understand cellular and molecular basis of periodontal tissue formation. This knowledge may provide an important platform to understand the mechanism of periodontal tissue development, and will be instrumental for effective stem-cell based regenerative therapies in the future.

## Supplementary Material

Sup fig1 **Supplementary Figure 1 ∣** scRNA-seq analysis of PTHrP-mCherry^+^ DF cells and Col1a1-GFP^+^ periodontal mesenchymal lineage cells. **(A)** FACS-sorting strategy for Col1a1-GFP^+^ cells (purple box), cells isolated from molars of *Col1a1(2.3kb)-GFP*; *PTHrP-creER*; *R26R-tdTomato* at P25 for scRNA-seq. **(B)** UMAP-based visualization of major classes of FACS-sorted cells. Feature plots of cell-type specific markers. High expression: violet, Low expression: yellow, No expression: gray, *n* = 8,630 cells from two datasets, 4,052 PTHrP-mCherry^+^ cells and 4,578 Col1a1-GFP^+^ cells.

Sup fig2 **Supplementary Figure 2 ∣** scRNA-seq analysis of PTHrP-mCherry^+^ DF cells and Col1a1-GFP^+^ periodontal mesenchymal lineage cells after re-clustering. UMAP-based visualization of major classes of FACS-sorted cells after re-clustering in mesenchymal lineage cells. Feature plots of cell-type specific markers. High expression: violet, Low expression: yellow, No expression: gray.

sup fig3 **Supplementary Figure 3 ∣** *In vivo* validation of the expression of selected marker genes in unbiasedly identified populations. **(a)** DF: RNAscope *in situ* hybridization (brown) of *Foxf1* of the mandibular molars at P3 for DF. **(b)** PDL: The mandibular molars of *Scx-GFP* mice at P25. **(c)** Immunofluorescence of DMP1 of the mandibular molars at P25 for osteoblasts. DF: dental follicle, DP: dental pulp, HERS: Hertwig’s epithelial root sheath, PDL: periodontal ligament, C: cementum, AB: alveolar bone. Scale bars: 200 μm **(a–c)**, 50 μm (a’-c’, c”).

sup fig4 **Supplementary Figure 4 ∣** Gene expression of cementoblast and osteoblast. UMAP-based visualization of top 20 genes in cementoblast (Cluster 9) and osteoblast (Cluster 11). Feature plots of cell-type specific markers. High expression: violet, Low expression: yellow, No expression: gray.

## Data Availability

The datasets presented in this study can be found in online repositories. The names of the repository/repositories and accession number(s) can be found in the article/**Supplementary Material**.
