# Supplementary figures and images for: Single-Cell Transcriptomic Analysis Reveals Developmental Relationships and Specific Markers of Mouse Periodontium Cellular Subsets
Source: Front Dent Med. Author manuscript; Available in PMC 2021 Dec 28. (PMC8713353; doi:10.3389/fdmed.2021.679937)

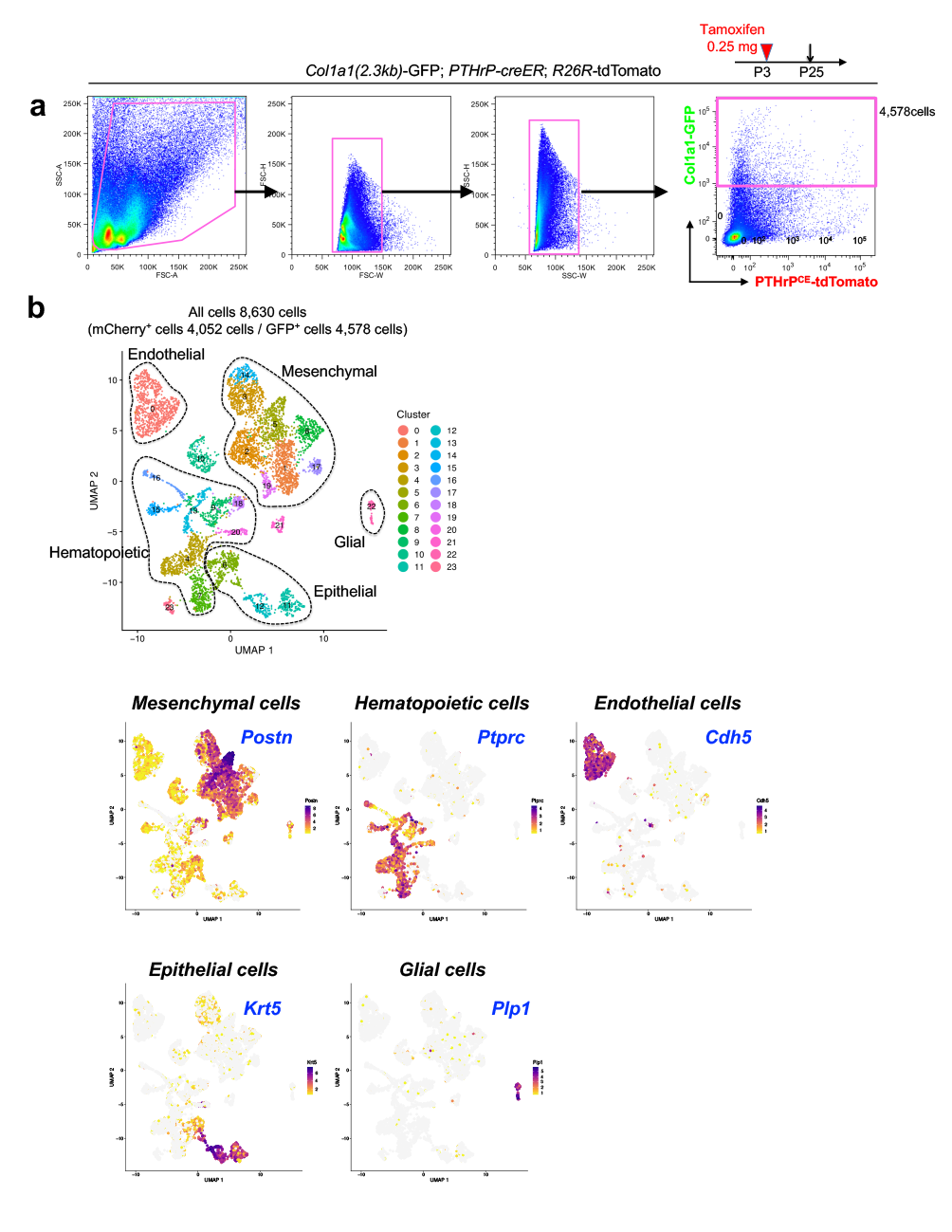

Supplement: Sup fig1 — Supplementary Figure 1 ∣ scRNA-seq analysis of PTHrP-mCherry+ DF cells and Col1a1-GFP+ periodontal mesenchymal lineage cells. (A) FACS-sorting strategy for Col1a1-GFP+ cells (purple box), cells isolated from molars of Col1a1(2.3kb)-GFP; PTHrP-creER; R26R-tdTomato at P25 for scRNA-seq. (B) UMAP-based visualization of major classes of FACS-sorted cells. Feature plots of cell-type specific markers. High expression: violet, Low expression: yellow, No expression: gray, n = 8,630 cells from two datasets, 4,052 PTHrP-mCherry+ cells and 4,578 Col1a1-GFP+ cells. [file NIHMS1763667-supplement-Sup_fig1.tif]

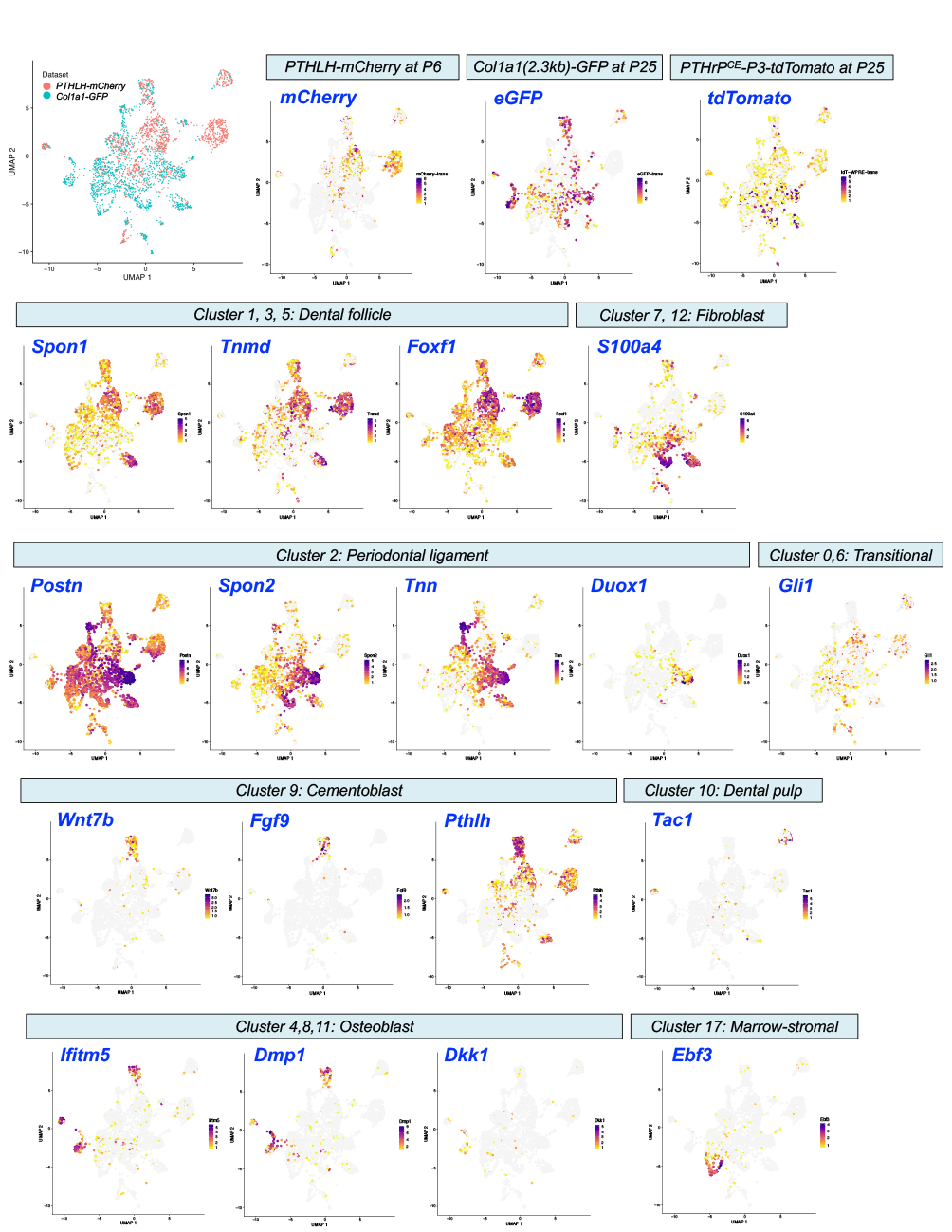

Supplement: Sup fig2 — Supplementary Figure 2 ∣ scRNA-seq analysis of PTHrP-mCherry+ DF cells and Col1a1-GFP+ periodontal mesenchymal lineage cells after re-clustering. UMAP-based visualization of major classes of FACS-sorted cells after re-clustering in mesenchymal lineage cells. Feature plots of cell-type specific markers. High expression: violet, Low expression: yellow, No expression: gray. [file NIHMS1763667-supplement-Sup_fig2.tif]

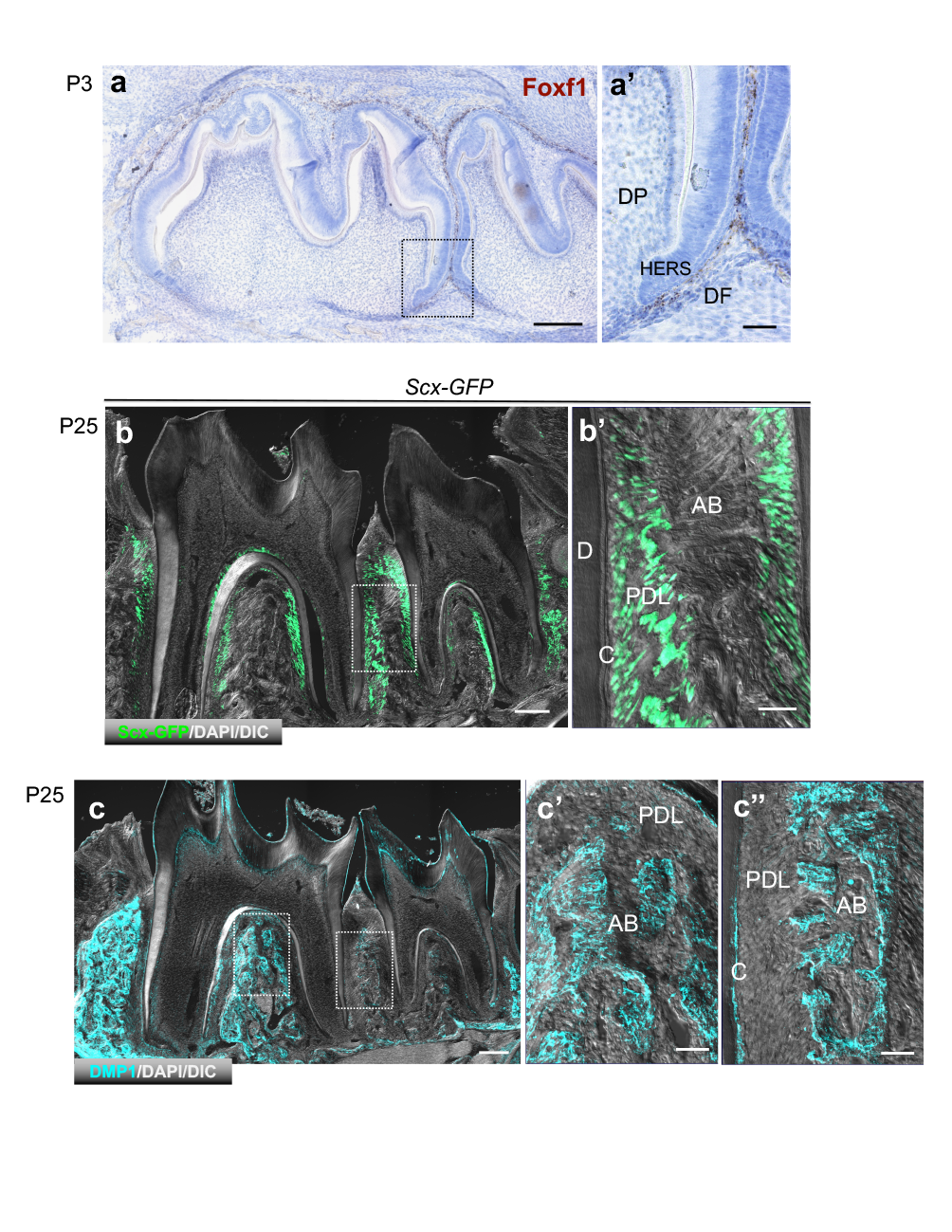

Supplement: sup fig3 — Supplementary Figure 3 ∣ In vivo validation of the expression of selected marker genes in unbiasedly identified populations. (a) DF: RNAscope in situ hybridization (brown) of Foxf1 of the mandibular molars at P3 for DF. (b) PDL: The mandibular molars of Scx-GFP mice at P25. (c) Immunofluorescence of DMP1 of the mandibular molars at P25 for osteoblasts. DF: dental follicle, DP: dental pulp, HERS: Hertwig’s epithelial root sheath, PDL: periodontal ligament, C: cementum, AB: alveolar bone. Scale bars: 200 μm (a–c), 50 μm (a’-c’, c”). [file NIHMS1763667-supplement-sup_fig3.tif]

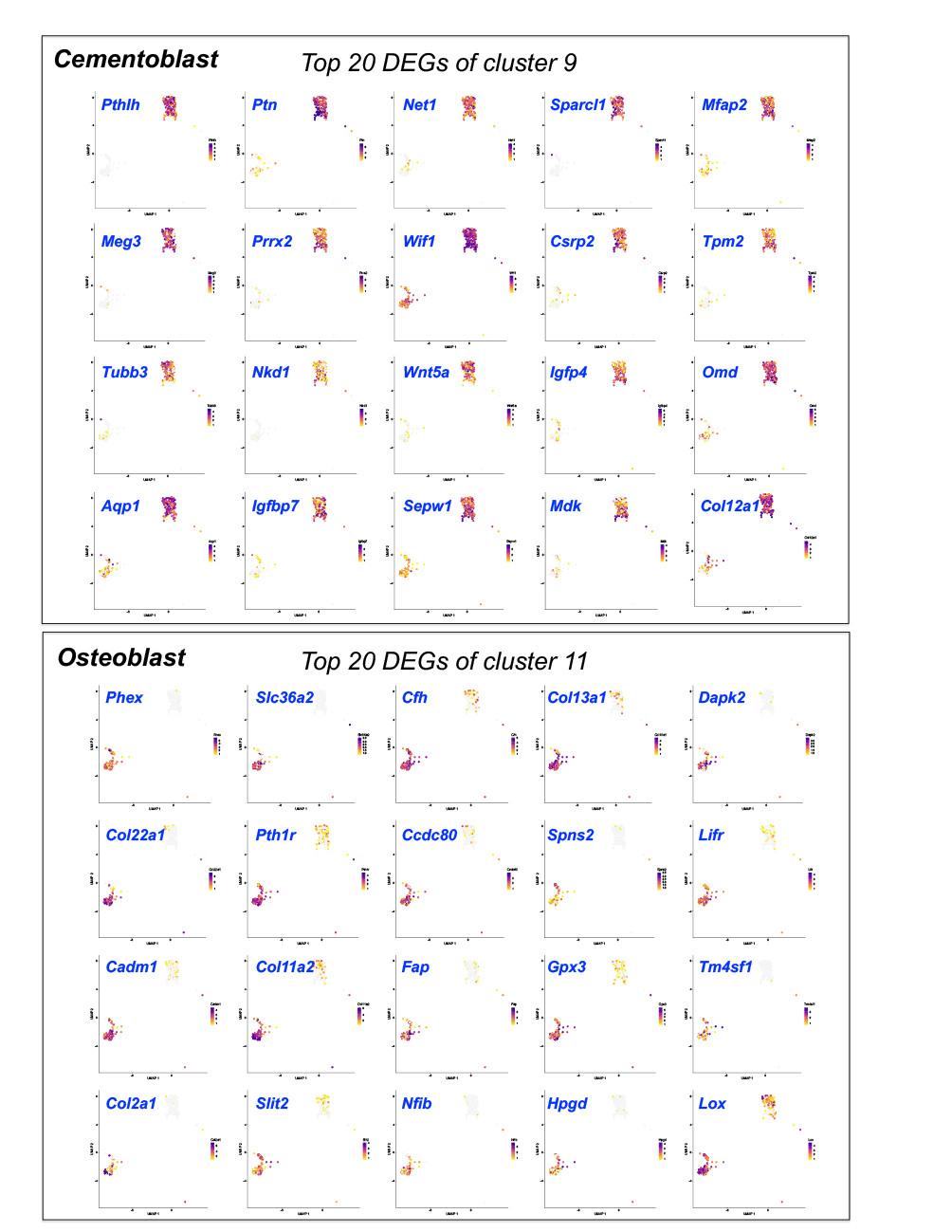

Supplement: sup fig4 — Supplementary Figure 4 ∣ Gene expression of cementoblast and osteoblast. UMAP-based visualization of top 20 genes in cementoblast (Cluster 9) and osteoblast (Cluster 11). Feature plots of cell-type specific markers. High expression: violet, Low expression: yellow, No expression: gray. [file NIHMS1763667-supplement-sup_fig4.tif]
